# A novel gene-based model for prognosis prediction of head and neck squamous cell carcinoma

**DOI:** 10.1016/j.heliyon.2024.e29449

**Published:** 2024-04-16

**Authors:** Yanxi Li, Peiran Li, Yuqi Liu, Wei Geng

**Affiliations:** a Department of Dental Implant Center, Beijing Stomatological Hospital, School of Stomatology, Capital Medical University, Beijing, 100050, China; b Department of Maxillofacial Surgery, Beijing Stomatological Hospital, School of Stomatology, Capital Medical University, Beijing, 100050, China

**Keywords:** Head and neck squamous cell carcinoma, Prognosis, Tight junction

## Abstract

**Background:**

Head and neck squamous cell carcinoma (HNSCC) is a significant global health challenge. The identification of reliable prognostic biomarkers and construction of an accurate prognostic model are crucial.

**Methods:**

In this study, mRNA expression data and clinical data of HNSCC patients from The Cancer Genome Atlas were used. Overlapping candidate genes (OCGs) were identified by intersecting differentially expressed genes and prognosis-related genes. Best prognostic genes were selected using the least absolute shrinkage and selection operator Cox regression based on OCGs, and a risk score was developed using the Cox coefficient of each gene. The prognostic power of the risk score was assessed using Kaplan-Meier survival analysis and time-dependent receiver operating characteristic analysis. Univariate and multivariate Cox regression were performed to identify independent prognostic parameters, which were used to construct a nomogram. The predictive accuracy of the nomogram was evaluated using calibration plots. Functional enrichment analysis of risk score related genes was performed to explore the potential biological functions and pathways. External validation was conducted using data from the Gene Expression Omnibus and ArrayExpress databases.

**Results:**

FADS3, TNFRSF12A, TJP3, and FUT6 were screened to be significantly related to prognosis in HNSCC patients. The risk score effectively stratified patients into high-risk group with poor overall survival (OS) and low-risk group with better OS. Risk score, age, clinical M stage and clinical N stage were regarded as independent prognostic parameters by Cox regression analysis and used to construct a nomogram. The nomogram performed well in 1-, 2-, 3-, 5- and 10-year survival predictions. Functional enrichment analysis suggested that tight junction was closely related to the cancer. In addition, the prognostic power of the risk score was validated by external datasets.

**Conclusions:**

This study constructed a gene-based model integrating clinical prognostic parameters to accurately predict prognosis in HNSCC patients.

## Highlights

• A prognostic signature based on four genes (FADS3, TNFRSF12A, TJP3, and FUT6) was constructed in HNSCC patients.
• A nomogram combining the gene prognostic signature and clinical phenotypes was established.
• The change in tight junction function was associated with the occurrence and development of HNSCC.

## Graphical abstract

**Figure:**
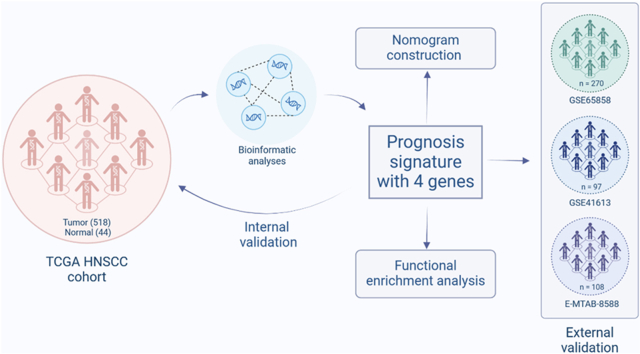

## 1 Introduction

Head and neck squamous cell carcinoma (HNSCC) develops from the mucosal epithelium in the oral cavity, pharynx and larynx. It is the most common malignancy that arises in the head and neck. Despite considerable efforts, from the period 1992–1996 to 2002–2006, the survival rate of HNSCC patients witnessed only marginal improvement, from 55 % to 66 % [1]. In the year 2020 alone, approximately 0.88 million new cases and 0.44 million new deaths for HNSCC was reported worldwide according to the Global Cancer Report, ranking eighth among all cancers [2]. Even worse, the incidence of this disease continues to rise unabated, with a projected escalation of 30 % by 2030, resulting in estimated 1.08 million new cases annually [3]. Besides the mortalities directly attributed to the disease, HNSCC patients experience a significantly elevated suicide rate of 63.4 cases per 100,000 individuals due to heightened psychological distress and impaired quality of life. Thus, the battle against HSNCC still has a long way to go.

Predicting the prognosis is crucial for cancer management and is still a challenge for many malignancies [4]. Traditional prognostic indicators such as tumor stages or grades exhibit limited precision in prognostication [5]. As we know, tumor development involves many genetic alterations. Technological advances have allowed changes in the expression of genes from tumor tissue resected from a patient to be detected and classified [6]. A gene expression signature is a single or a particular group of genes correlating genetic alterations with specific clinical variables, such as diagnosis, prognosis or prediction of the therapeutic response [7]. Prognostic gene expression signatures can help improve patients’ therapy by classifying tumors into separate groups, thus providing guidance for a personalized treatment-decision. In breast cancer, multi-gene prognostic tools are commercially available, and the clinical use is mature, allowing optimized use of the current therapeutic resources by reducing the rates of over/under treatment and avoiding unnecessary side-effects in cancer patients [8]. In the field of head and neck cancer, various prognostic models have been developed. Unfortunately, to different degrees, these models have limitations in terms of ease of use, accuracy, and applicability to specific patient populations. Thus, none of them have not been implemented in the clinical routine yet, or even entered preclinical and clinical investigations. We found that existing studies largely constructed prognosis signatures based on a specific group of genes (like immune-related gene [9], metabolic enzyme-based genes [10]), which means other genes were excluded. In this context, we would like to construct a gene signature based on the full transcriptome, rather than a set of genes performing certain functionality.

In this study, we obtained mRNA expression data and clinical data of HNSCC patients from four independent datasets, with The Cancer Genome Atlas (TCGA) as the training set and GSE65858, GSE41613, E-MTAB-8588 as the validation sets. A prognosis gene signature based on four genes was constructed and validated. The risk score was calculated through the multivariate Cox coefficient multiplied by the expression of the gene. Then a nomogram was established combning the risk score and clinical parameters. Such a model would enable accurate prediction and facilitate the customization of prevention, screening, and treatment strategies for individuals with HNSCC. Finally, functional enrichment analysis was performed to identify the potential biological functions and pathways of the genes related to the risk score.

## 2 Materials and methods

### 2.1 Acquisition and preprocessing of data

The mRNA expression data and clinical information pertaining to the training cohort were retrieved from The Cancer Genome Atlas (TCGA) database (https://tcga-data.nci.nih.gov/tcga/). Information of the validation groups was downloaded from ArrayExpress database (https://www.ebi.ac.uk/biostudies/arrayexpress) and Gene Expression Omnibus (GEO) database (https://www.ncbi.nlm.nih.gov/geo/). Patients lacking information on survival time (FU time, follow up time or OS, overall survival) or survival status were excluded from the analysis. Immunohistochemistry (IHC) data concerning HNSCC and normal tissues were obtained from the Human Protein Atlas (HPA) portal (https://www.proteinatlas.org/).

### 2.2 Identification of candidate genes

The selection of candidate genes was conducted by overlapping differentially expressed genes (DEGs) and genes associated with prognosis. To identify DEGs, a comparison of gene expression levels was conducted between tumor and paracancerous groups. DEGs were defined as genes with a p-value <0.001 and |log_2_ (Foldchange)| > 1.58, which means Foldchange ≥3 (upregulation) or Foldchange ≤1/3. Prognosis-related genes were identified using log-rank tests and univariate Cox proportional hazards regression analysis. Genes with an Hazard Ratio (HR) > 1 and a p-value <0.01 were considered as risky prognostic genes, while genes with an HR < 1 and a p-value <0.01 were considered as protective prognostic genes. The risky prognostic genes were then intersected with the upregulated DEGs, while the protective prognostic genes were intersected with the downregulated DEGs, resulting in overlapping candidate genes (OCGs) for subsequent analysis.

### 2.3 Development of a multi-gene prognostic signature

To establish a prognostic signature, we used the Cox proportional hazard model with the least absolute shrinkage and selection operator for variable selection (LASSO-Cox), which is suitable for the regression of high-dimensional data, through the glmnet and survival packages in R [11,12]. The λ value corresponding to the minimum partial likelihood deviance was selected as the optimal λ for this study.

The expression level of the optimal prognostic genes in tumor and normal samples was compared using Mann Whitney test since the data did not meet the normality requirement. To validate the expression profile in protein level, IHC staining pictures of the optimal prognostic proteins in normal tissues and HNSCC tissues were downloaded from the HPA portal. Patients were divided into two groups (high expression group and low expression group) evenly based on the expression level the optimal prognostic genes. Kaplan-Meier (KM) survival analysis with log-rank test was conducted to test the prognostic value of the optimal prognostic genes. In addition, patients were divided into two groups unevenly, and P-value based on different grouping ways were calculated.

The risk score for each patient was calculated using the following formula:

$$\text{risk}\;\text{score}=\sum_{i=1}^{4}\lambda i\times \text{Expi}$$

where λi represents the corresponding λ value, and Expi represents the gene expression level (fpkm) of each gene.

### 2.4 Construction and validation of the nomogram

Univariate and multivariate Cox regression were performed to identify independent prognostic parameters. Then, the nomogram analysis was conducted in the training group using the rms package in R [13]. The nomogram consists of an upper part representing the scoring system and a lower part representing the prediction system. By assigning total points based on the sum of points for each factor, the nomogram could predict the 1-, 2-, 3-, 5-, and 10-year survival rate of HNSCC patients. C-Index values and calibration curves were utilized to demonstrate the accuracy of the survival prediction [14].

### 2.5 Functional enrichment analysis

The correlation between the risk score and the expression levels of all genes in tumor patients was assessed using the Pearson correlation coefficient [15]. Genes with a Pearson's coefficient below −0.4 or above 0.4 were identified as risk score-related genes. Subsequently, Gene Ontology (GO) and Kyoto Encyclopedia of Genes and Genomes (KEGG) pathways analyses of risk score-related genes were conducted using the DAVID portal website (https://david.ncifcrf.gov/summary.jsp). A p-value below 0.05 was considered statistically significant. To determine the biological functional enrichment score for each patient, Gene Set Variation Analysis (GSVA) was performed utilizing the tumor transcriptome sequencing data. The gsva package in R was employed to conduct the GSVA analysis with default parameters [16]. The gene lists for each biological function were obtained most recently from the GSEA Web portals (GSEA | Login (gsea-msigdb.org)).

### 2.6 External validation of the multi-gene prognostic signature

In the external validation sets, the risk score for each patient was calculated using the same methodology as the training group. Subsequently, the patients were categorized into high-risk and low-risk groups based on the median risk score obtained from the validation set. The performance of the multi-gene prognostic signature was validated using KM survival analysis with log-rank test and time dependent receiver operating characteristic (ROC) analysis. The area under the ROC curve (AUC) was calculated to make a comparison for discriminatory ability of above prognostic parameters [17].

### 2.7 Statistical analysis

Statistical analyses were conducted using R (https://www.r-project.org/, v4.3.0), SPSS software (IBM, v25.0). GSEA analyses were performed using the GSEA package in java software (http://software.broadinstitute.org/gsea/index.jsp). A significance level of P < 0.05 was considered statistically significant if not stated otherwise.

## 3 Results

### 3.1 Establishment of the prognostic signature

**Fig. 1:**
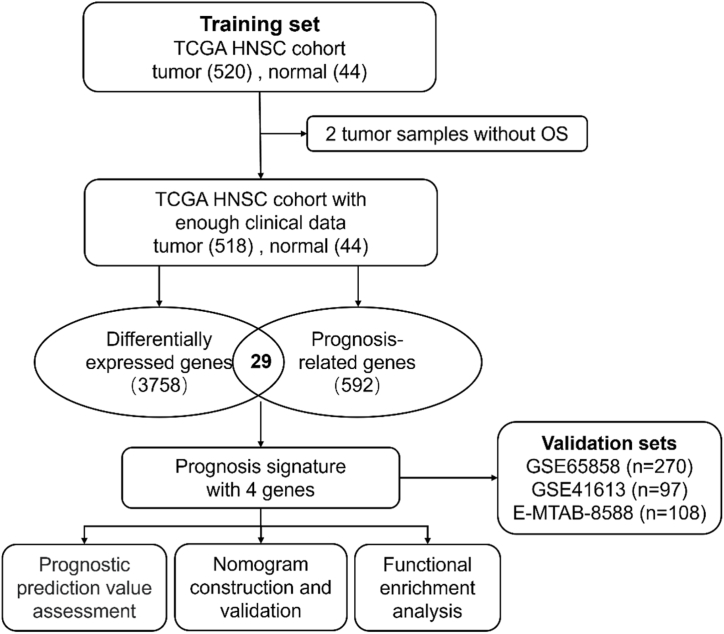
Flowchart of this study.

**Fig. 2:**
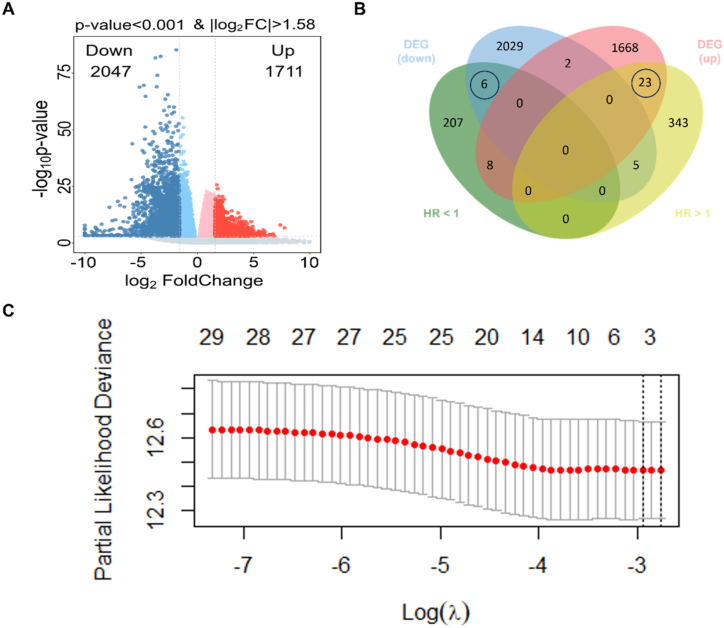
Establishment of the prognostic signature. (A) Differentially expressed genes shown in a volcano plot (tumor vs normal). Red in the plot indicates upregulation, and blue indicates downregulation. (B) By intersecting differentially expressed genes and prognosis-related genes, a total of 29 overlapping candidate genes were identified. 23 upregulated genes with HR > 1 and 6 downregulated genes with HR < 1. (C) Screening the most representative 4 genes in 29 overlapping candidate genes by LASSO‐Cox analysis.

The research flowchart is presented in Fig. 1. In the training set, mRNA expression data and clinical information of 564 samples (consisting of 520 tumor samples and 44 paracancerous samples) from HNSCC patients were obtained from TCGA dataset. Two samples from the tumor group were excluded due to the lack of OS data. By comparing the gene expression levels between 518 tumor samples and 44 paracancerous samples, 1711 upregulated and 2047 downregulated DEGs were identified (Fig. 2A). A total of 592 prognosis-related genes were identified, including 371 risky and 221 protective prognostic genes. By intersecting these genes, a total of 29 OCGs were identified (Fig. 2B). Subsequently, based on these OCGs, 4 optimal prognostic genes (FADS3, TNFRSF12A, TJP3, and FUT6) and their corresponding λ values (FADS3: 0.00247280389163646, TNFRSF12A: 0.0000481768472410848, TJP3: 0.00258138107120143, and FUT6: 0.002883491941) were determined (Fig. 2C).

### 3.2 Expression profiles of four optimal prognostic genes

**Fig. 3:**
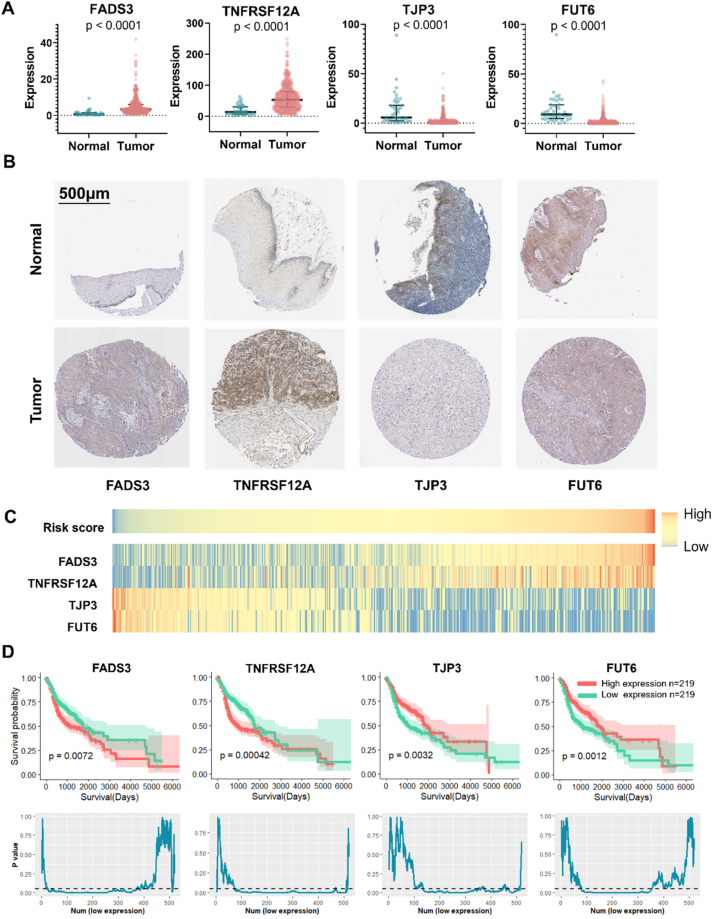
Expression profiles of four optimal prognostic genes. (A) The expression level of the optimal prognostic genes in tumor and normal samples was compared using Mann Whitney test and presented by median (interquartile range). (B) IHC staining of the four proteins in normal tissue and HNSCC tissue. (C) Heatmap of the expression profiles of the four prognostic genes in patients. (D) KM survival analysis of FADS3, TNFRSF12A, TJP3 and FUT6. The line chart showed the P values of survival analysis between patients in low- or high-expression group with various cutoff.

The expression profiles of the four optimal prognostic genes between tumor and normal tissue are depicted in Fig. 3A. FADS3 and TNFRSF12A were found to be significantly upregulated, while TJP3 and FUT6 were significantly downregulated in HNSCC samples compared to normal samples. Furthermore, the protein expression of these four genes was validated through IHC staining results obtained from the Human Protein Atlas database (Fig. 3B). The λ values were utilized to calculate the risk score for each patient. Fig. 3C displays the risk scores of 518 patients in the training database. Based on the median value (0.005892238), patients were categorized into high- and low-risk groups. The expression level of FADS3 and TNFRSF12A was higher in the high-risk group compared to the low-risk group, whereas TJP3 and FUT6 exhibited the opposite expression pattern (Fig. 3C). This indicates that FADS3 and TNFRSF12A are considered risky prognostic genes, while TJP3 and FUT6 serve as protective prognostic genes. Furthermore, the prognostic predictive value of the four optimal prognostic genes in HNSCC patients was explored. KM analysis was conducted based on the training database. 518 patients were divided into high expression and low expression group evenly based on the expression level of FADS3, TNFRSF12A, TJP3 or FUT6. Overexpression of FADS3 or TNFRSF12A was associated with poor prognosis, whereas patients with low expression of TJP3 or FUT6 exhibited significantly shorter OS compared to those with higher expression (Fig. 3D). Besides, we divided patients into two groups unevenly, with abscissa represents the number of people included in the low expression group. These conclusions remained consistent even with different arbitrary cutoffs (Fig. 3D). These results collectively indicate the prognostic predictive value of the four optimal prognostic genes in HNSCC patients.

### 3.3 The risk signature can stably predict the prognosis of HNSCC patients

**Fig. 4:**
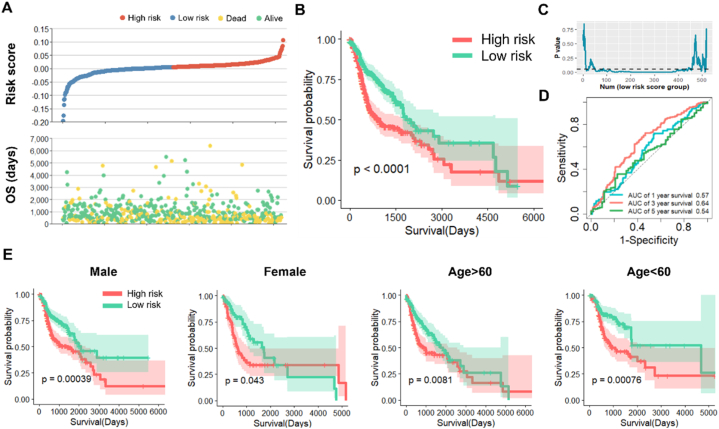
The risk signature can stably predict the prognosis of HNSCC patients. (A) The curve of risk score and survival status of the patients. Dots share the same abscissa represents one individual. More dead patients corresponding to the higher risk score. (B) Kaplan–Meier survival analysis of the four-gene signature. (C) P values of survival analysis between patients in low or high-risk group with various cutoff. (D) Time-dependent ROC analysis the of the four gene signature. The AUC value is used to assess the accuracy of prediction. (E) Kaplan–Meier survival analysis in different subgroups including male, female, older than 60 years old and younger than 60 years old. In KM survival curves, the horizontal axis and the vertical axis are time and survival rates, respectively. Red color represents high risk group and green color represents low risk group.

We thereafter evaluated the prognostic significance of risk score. As shown in Fig. 4A, the risk of death increased with an increase of risk score. Survival curves were generated by KM survival analysis. The results demonstrated a significantly worse prognosis for patients in the high-risk group in comparison to those in the low-risk group (Fig. 4B). The conclusion remained consistent with different arbitrary risk value cutoffs (Fig. 4C). The AUC values were up to 0.57, 0.64 and 0.54 at 1-, 2- and 3-year respectively in ROC analysis (Fig. 4D). Furthermore, we assessed the predictive power of the risk score in different clinical subgroups (Fig. 4E). The KM survival curves suggested that patients with high-risk scores exhibited worse OS compared to patients with low-risk scores in subgroups stratified by gender (male and female) as well as age (>60 and < 60). All results above confirmed the predictive accuracy of our risk signature.

### 3.4 The personalized prediction model showed robust predictive accuracy

**Fig. 5:**
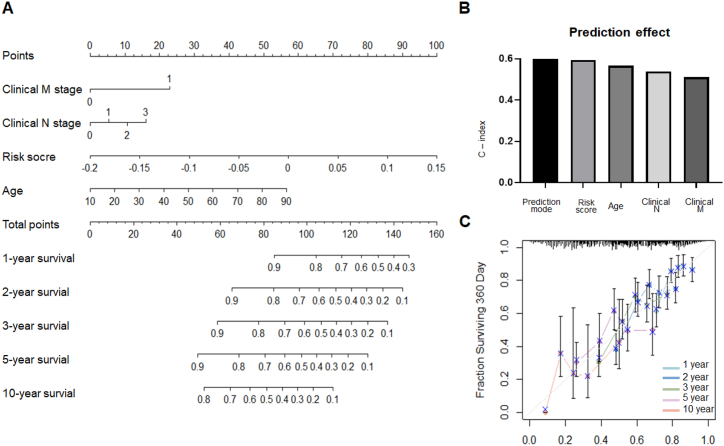
Construction of gene-based prognostic model. (A) Nomogram integrated 4 gene-based risk score, clinical M stage, clinical N stage and age. The 1‐, 2‐ 3‐, 5‐, and 10‐year survival rate of HNSCC patients could be predicted by the nomogram. (B) The predictive effect of the individualized prediction model, risk score, age, clinical N stage and clinical M stage was evaluated by C‐Index. (C) The calibration plot of the nomogram for agreement test between predicted and actual outcome in the training set. X and y axes represent survival rates estimated by nomogram and the actual survival rates, respectively.

Univariate and multivariate Cox proportional hazards regression were performed to identify independent prognostic variables of the OS in the training set. The results revealed that risk score, age, clinical M stage and clinical N stage could serve as independent prognostic factors for OS (Table 1). To enhance the clinical applicability of the prognostic prediction model, an individualized prediction model was developed (Fig. 5A), incorporating the independent predictive factors mentioned above. The C-index of this nomogram model was 0.78, surpassing that of any other prediction model (Fig. 5B). Additionally, the calibration curve demonstrated a satisfactory alignment between the nomogram and actual observations, indicating an optimal level of predictive accuracy (Fig. 5C).

**Table 1:** Univariate and multivariate analysis of prognostic parameters in HSNCC.

Variable	Univariate analysis				Multivariate analysis			
	Exp(B)	95.0 % CI for Exp(B)		*P*‐value	Exp(B)	95.0 % CI for Exp(B)		*P*‐value
		Lower	Upper			Lower	Upper	
Risk score	18793.489	56.116	6294070.905	0.001	46760.816	107.543	20332104.093	**0.001**
Age	1.025	1.012	1.038	0.000	1.028	1.014	1.042	**0.000**
Clinical M	0.288	0.107	0.778	0.014	3.168	1.100	9.123	**0.033**
Clinical N	1.128	0.973	1.309	0.110	1.326	1.072	1.638	**0.009**
Clinical T	1.126	0.975	1.301	0.107	1.266	0.970	1.651	0.082
Clinical stage	1.104	0.945	1.289	0.212	0.803	0.573	1.126	0.204
Alcohol history	1.043	0.780	1.396	0.775	1.020	0.749	1.388	0.901
Gender	1.340	0.997	1.803	0.053	0.930	0.671	1.288	0.661

CI, confidence interval.

### 3.5 The risk score exhibits a strong association with tight junction

**Fig. 6:**
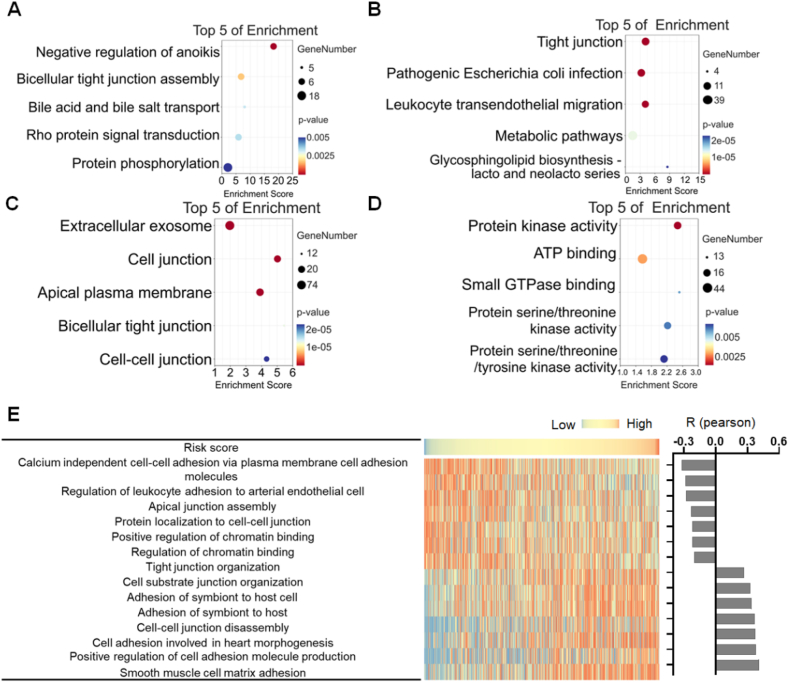
Functional enrichment analysis of risk score related DEGs. (A) Biological processes, (B) cellular components, and (C) molecular functions that were mostly related to risk score. (D) KEGG pathway analysis of genes most related to risk score. (E) Correlation analysis between risk score and cell junction related function enrichment scores. The column graph on the right showed the R‐value of the correlation analysis.

To investigate the biological functions and pathways associated with the risk score, the GO enrichment analysis and KEGG analysis were performed based on the genes most related to the risk score. The results indicated that the risk score was significantly correlated with cell junction, especially tight junction (Fig. 6A–D). Therefore, GSVA analysis was performed to determine the enrichment score of cell junction related processes. The results revealed that some cell junction related processes were positively while some were negatively correlated with risk score (Fig. 6E). These results suggested that the change in cell junction might play a role in occurrence and development of HNSCC.

### 3.6 External validation of the prognostic signature

**Fig. 7:**
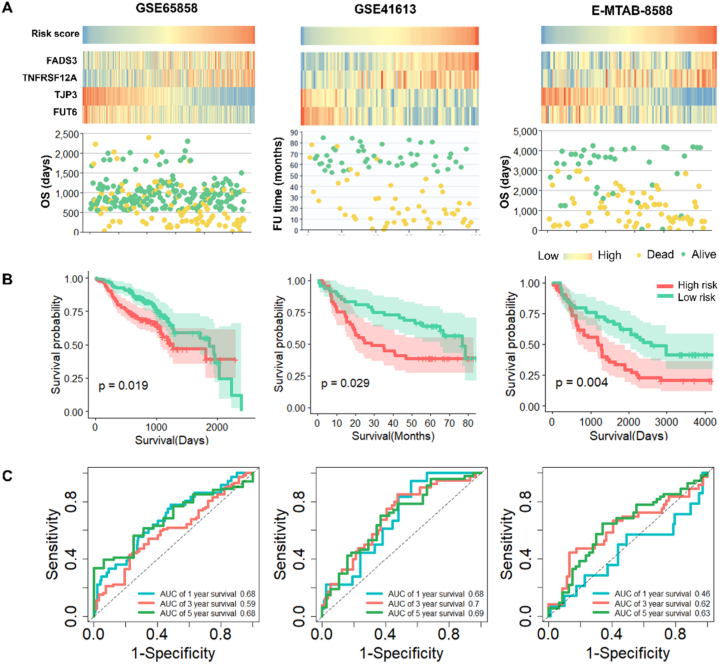
External validation of the prognostic signature. (A) Heatmap of risk score and the expression profiles of the four prognostic genes in patients. Survival status of the patients. More dead patients corresponding to the higher risk score. Squares and dots share the same abscissa represents one individual. (B) Kaplan–Meier survival analysis of the four gene signature in validation sets. (C) Time-dependent ROC analysis of the four gene signature in validation sets. The AUC value is used to assess the accuracy of prediction.

The prognostic prediction performance of risk score was further verify based on two external validation datasets (GSE29609, n = 270 and E-MATB-8588, n = 108). The risk score for each patient in validation datasets was calculated, and patients were classified into high‐ and low‐risk groups based on median of risk score (Fig. 7A). The expression patterns of FADS3, TRSF12A, TJP3, and FUT6 in the two validation sets were found to be similar to those observed in the training set. Furthermore, a higher risk score was associated with an increased risk of mortality (Fig. 7A). Additionally, the KM curves of the two validation sets demonstrated that the high-risk group had a significantly worse prognosis compared to the low-risk group (Fig. 7B). Time-dependent ROC analysis showed that AUC for 1-, 3-, and 5-year OS of the external validation sets were 0.68, 0.59, 0.68, 0.68, 0.7, 0.69, 0.46, 0.62 and 0.63, respectively (Fig. 7C). To sum up, the prognostic signature exhibited favorable performance in predicting the overall survival of HNSCC patients.

## 4 Discussion

HNSCC presents a substantial challenge for humanity. Conventional prognostic models based on single clinical parameters have limited predictive power. Integrating bioinformatics and clinical information offers a promising approach to enhance prediction accuracy. In this study, by taking the intersection between DEGs and prognosis related genes, we selected candidate genes that are most likely to modulate tumor growth either positively or negatively. Subsequently, a risk prediction model consisting of four genes (FADS3, TNFRSF12A, TJP3, and FUT6) was established based on these candidates. The expression levels of these 4 genes were significantly different between normal and cancer tissues and were also associated with OS of HNSCC patients. Moreover, the prognostic value of this four-gene prognostic signature in HNSCC patients was investigated. The patients classified into high-risk group exhibited substantially worse prognoses compared to those in the low-risk group. In addition, the predictive value of the four-gene prognostic signature was consistent across different subgroups, including male, female, age >60, and age <60 subgroups. Besides, this gene model can effectively stratify HNSCC patients in four external datasets. Then, a novel nomogram was developed to predict survival probability in HNSCC patients. This nomogram, incorporating the risk score, age, clinical M stage and clinical N stage, demonstrated successful identification of patients. At last, biological functions analysis based on the genes most related to the risk score showed that cell junction, especially tight junction might play a role in the occurrence and development of HNSCC.

In fact, gene signatures predicting the prognosis of HNSCC have been established in previous studies. For example, an NK cells-related gene signature was reported to perform well in assessing the prognosis of HNSCC patients [18]; an oxidative stress-related gene signature might predict prognosis in HNSCC patients [19]; a prognostic signature based on autophagy, apoptosis and pyroptosis-related genes was constructed [20]. However, these existing signatures were developed by analyzing small numbers of specific genes. As we know, genes with distinct functions (e.g., angiogenesis [21], metabolism [22], immune escape [23]) have previously been implicated in cancer development, rather than a specific group of genes with specific functions. Therefore, in this study, we recognized DEGs based on all genes, rather than a specific set of genes.

The four genes included in the prognostic signature displayed significant associations with the OS of HNSCC patients. Specifically, FADS3 and TNFRSF12A were identified as risk prognostic genes, whereas TJP3 and FUT6 were deemed protective genes. FADS3, as a member of the fatty acid desaturase family, has received increasing attention in tumor biology [[24], [25], [26]]. Indeed, alterations in lipid metabolism in cancer are recognized. De novo fatty acid synthesis is heightened in tumors to sustain cell proliferation and tumor growth, because lipids are not only components of biological membranes, but also play important roles in the process of signal transduction [27]. FADS3 encodes an enzyme that catalyzes double bond introduction into the fatty acid acyl chains (a chemical modification that determines the level of phospholipids packing), therefore regulating cell membrane fluidity and dissemination of cancer cells [25]. In breast cancer, FADS3 has been observed to enhance cell membrane fluidity and facilitate hematogenous diffusion and lung metastasis [25]. In HNSCC patients, consistent with our findings, a study by Su et al. indicated that elevated expression of FADS3 was related to higher lymphatic metastasis, higher histologic grade, lymphovascular invasion and unfavorable prognosis [20]. Besides, the author found that FADS3 was related to the inhibition of amino acid metabolism and reduced levels of B cells [20].

TNFRSF12A, alternatively named FN14, belongs to the TNF/TNFR superfamily and has been reported to be involved in the initiation and progression of multiple tumor types, including glioma, pancreatic, breast, non-small-cell lung cancer, and colorectal cancer [[28], [29], [30]]. Inhibition of TNFRSF12A has been found to attenuate cancer-related cachexia and extend patient survival [31]. Previous studies demonstrated the role of TNFRSF12A in oral squamous cell carcinoma. TNFRSF12A was highly expressed in tumors. Besides, it expressed significantly higher at the invasive tumor front than in the whole tumor [32]. As reported, mechanistically, high expression of TNFRSF12A stimulates cell migration and invasiveness. In addition, it can promote the expression of FGF-2 and VEGF, which further promote angiogenesis and tumor progression [30].

TJP3, also known as ZO-3, functions as a scaffolding protein that indirectly connects membrane tight junction proteins to the actin cytoskeleton and cell signaling pathways [33]. In breast cancer, the level of ZO-3 was lower in tumor tissues compared with normal tissues. Besides, levels of ZO-3 were reduced with increasing TNM status [34]. In this study, decreased ZO-3 was found in the tumor group, suggesting that tight junction damaged by downregulation of ZO-3 might promote the cancer metastasis. However, in some other studies, upregulated ZO-3 was reported to promote cancer [35,36]. Future studies are needed to investigate the exact role ZO-3 played in cancer.

FUT6 belongs to the fucosyltransferas family and is responsible for fucosylation synthesis. A recent study revealed that FUT6 suppresses the proliferation, migration, invasion, and EGF-induced epithelial-mesenchymal transition in HNSCC cells [37], which corroborated our findings. In addition, in another signature based on metabolic enzymes, FUT6 was also identified as a protective biomarker [10]. As reported, high expression of FUT6 is related to the occurrence and metastasis of a wide range of cancer types, including breast cancer [38], gastric cancer [39] and colorectal cancer [40]. However, the role of fucosyltransferas in different tumors were not the same [41].

In order to improve the ability to prognosis prediction of gene prognostic signature, a nomogram, incorporating the risk score, age, clinical M stage and clinical N stage, was developed. Perfect agreement between the predicted and observed outcomes indicating the high precision of our nomogram in prognosis prediction. Using this nomogram, 1-, 2-, 3-, 5- and 10- year OS probability of a patient can be predicted according to the risk score and other conventional clinical prognostic parameters, which assist both physicians and patients in decision making.

To investigate the biological functions and pathways associated with the risk score, genes most related to the risk score were identified and the GO enrichment analysis and KEGG analysis were constructed based on them. The results indicated that the risk score was significantly correlated with cell junction, especially tight junctions. Tight junctions are epithelial intercellular junctions located at the apical region of cell– cell contact [42]. Basically, tight junction proteins are responsible for regulating paracellular permeability and maintaining cell polarity [43]. Recently, research has revealed that tight junction proteins are not merely static constituents of cell junctions but rather multifunctional signaling complexes involved in the regulation of various cellular processes [44]. Changes in the expression and localization of these molecules are frequently observed in cancer, implying their potential roles in cancer development. Tight junction proteins have been implicated in a wide range of cellular events critical to the initiation and progression of cancer, including proliferation, migration, plasticity, and differentiation [45]. They have been identified as potential mediators of apoptosis/anikiosis resistance, acquisition of a cancer stem-like phenotype, collective cell migration, and invasive cellular behavior [46]. However, the involvement of tight junction proteins in HNSCC remains largely unexplored. In this research, functional enrichment analysis indicated that the prognostic signature is mainly associated with tight junctions. This suggests that further study could focus on elucidating the effects and underlying mechanisms of tight junctions in HNSCC, to understand how molecular abnormalities in the expression of tight junction proteins could contribute to tumorigenesis, and to expand their use as tools for cancer diagnosis, prognosis and treatment.

Despite providing a gene signature and a reliable prediction model that integrates both bioinformatics and clinical information, this study has several limitations that need to be acknowledged. Firstly, the molecular mechanisms underlying the influence of the identified four genes and tight junctions on HNSCC were not investigated in this study, which can be the direction of further studies. Secondly, this study exclusively focused on the mRNA sequencing data and did not consider other types of data, such as single nucleotide polymorphisms (SNPs), copy number variations (CNVs), and DNA methylation. In the future, all kinds of bioinformation would be analyzed to obtain a more comprehensive conclusion. Thirdly, clinical studies with a large sample size are needed to further validate those findings.

## 5 Conclusions

In summary, the present study constructed a prognostic signature based on four genes (FADS3, TNFRSF12A, TJP3, and FUT6). Besides, a nomogram combined clinical phenotype–gene prognostic signature was established, which showed high predictive efficacy and can be used to predict HNSCC patient prognostic risk in the clinical setting. In addition, the prognostic signature is mainly associated with tight junctions, suggesting future research directions.

## Funding

This work was supported by Beijing Stomatological Hospital, 10.13039/501100002799Capital Medical University Young Scientist Program (No. YSP202209) and 10.13039/501100001809National Natural Science Foundation of China (No. 62071313).

## Data availability

The datasets presented in this study can be found in online repositories. The names of the repository/repositories and accession number(s) can be found in the article.

## CRediT authorship contribution statement

**Yanxi Li:** Data curation, Formal analysis, Funding acquisition, Investigation, Resources, Writing – review & editing. **Peiran Li:** Methodology, Software, Validation. **Yuqi Liu:** Writing – original draft. **Wei Geng:** Conceptualization, Funding acquisition, Project administration, Supervision.

## Declaration of competing interest

The authors declare that they have no known competing financial interests or personal relationships that could have appeared to influence the work reported in this paper.
